# Prognostic factors and predictors of outcome in children with autism spectrum disorder: the role of the paediatrician

**DOI:** 10.1186/s13052-021-01008-5

**Published:** 2021-03-18

**Authors:** Magda Di Renzo, Federico Bianchi di Castelbianco, Villani Alberto, Del Vecchio Antonio, Corsello Giovanni, Elena Vanadia, Massimiliano Petrillo, Trapolino Davide, Lidia Racinaro, Monica Rea

**Affiliations:** 1 Institute of Ortofonologia (IdO), Via Salaria 30, 00198 Rome, Italy; 2 Institute of Ortofonologia (IdO), Via Tagliamento 25, 00198 Rome, Italy; 3 Società Italiana di Pediatria, Rome, Italy; 4 Dipartimento Emergenza Accettazione Pediatria Generale, IRCCS – Ospedale Pediatrico Bambino Gesù, Rome, Italy; 5 Terapia Intensiva Neonatale e Neonatologia, Ospedale Di Venere, Bari, Italy; 6 Clinica Pediatrica Università di Palermo, Palermo, Italy

**Keywords:** Autism spectrum disorder, Predictors, Outcome, Paediatrician, Emotional variables

## Abstract

**Background:**

Autism spectrum disorder is a complex condition with wide variation in type and severity that involves persistent challenges in social interaction, speech and nonverbal communication, restricted/repetitive behaviours and adaptive behaviours. In recent years, research has deepened the study of the predictive factors of optimal outcome, intended as indicators of positive trajectory in children with a previous diagnosis of autism who, after a therapeutic path, show a significant reduction in the “core” symptoms of autism and a positive evolution in social, adaptive, affective, and relational skills.

**Methods:**

The study included 40 children aged 21 to 66 months, enrolled between 2015 and 2016 for an autism spectrum disorder clinical suspicious. Children were re-evaluated after at least 2 years of therapy and they were divided into two groups: the ASD-ASD group included children with a confirmed diagnosis of ASD, and the ASD-OO comparison group included children who no longer met the criteria for an autism classification. The aim of this retrospective study was to investigate the presence of cognitive, emotional and relational predictors capable of predicting the presence of optimal outcome in with a diagnosis of autism; the predictors taken into consideration were the intelligence quotient, the play, the emotional contagion and the understanding of other’s intentions. In this way, it is possible to support clinicians in defining a more complete diagnostic framework of autism, using assessment tools that can be administered quickly and therefore suitable for short observation sessions in paediatric patients.

**Results:**

The findings showed that 15 out of 40 children, after at least for 2 years, no longer fell into the diagnostic ASD category based on the ADOS-2, DSM-5 and clinical criteria. The children in the ASD-OO group initially had a higher IQ than those in the ASD-ASD group, lower severity of autistic symptoms, greater understanding of intentions, more emotional contagion, and better quality of play. The results suggest that the initial coexistence of skills in these areas at the time of the first diagnostic assessment may allow us to predict the possibility of achieving optimal outcome after 2 years of therapy.

**Conclusions:**

The data of this study highlight the importance of considering, during assessment, intelligence quotient, play, emotional contagion, and understanding of the intentions of others as potential prognostic predictors that can become useful tools for clinicians and paediatricians. This allows us to focus attention, in both the diagnostic and prognostic phases, on emotional-relational variables that can support the clinician in defining a more complete diagnostic framework and in planning a more personalized therapeutic path.

## Introduction

Autism spectrum disorder (ASD) is considered a complex condition with different levels of severity that is characterized by impairments in the social, communicative, affective, and adaptive spheres. The characteristics of clinical symptoms can be very heterogeneous in terms of both complexity and severity. Furthermore, individuals with ASD often have different neurological, psychiatric, and medical comorbidities. International epidemiological studies report a generalized increase in the prevalence of ASD: currently, the median value of the prevalence is 6.2/1000 (1: 160; range: 1: 333–1:86) in Europe and 6.5/1000 (1: 154; range: 1: 769–1:91) in the USA, with great variability in estimates between and within geographical areas. In the latest report published by the Centers for Disease Control and Prevention [1], it is estimated that approximately 1 in 68 (14.6 per 1000) school-aged children have an autism spectrum disorder with a clear male prevalence (4.5: 1).

No differences emerge in the prevalence of ASD between America, the Western Pacific area and Europe [2], and there are no official data referring to the African continent.

There are no national prevalence estimates in Italy, and the only prevalence estimates available in Italy refer to the Emilia-Romagna and Piemonte regions. The prevalence in Piemonte is 5% in the 7–11 age group, and in Emilia-Romagna, it is 3.9% in the 0- to 17-year age group (distribution by age group: 0–2 years: 2.9%; 3–5 years: 5.5%; 6–10 years: 4.3%; 11–17 years: 3%).

In recent years, research inquired into the presence of any predictive factors of optimal outcome (OO), defined as a positive trajectory in children with a prior diagnosis of ASD who, following a therapeutic path, showed not only a significant reduction in the “core” symptoms of autism but also a positive evolution of social, adaptive, affective, and relational skills. As described by Fein et al. [3], the first definition of “best outcome” or “recovery from autism” dates to the late 1980s, when Lovaas [4] found that 47% of children diagnosed with autism, after being involved in intensive behavioural intervention, were able to attend primary school regularly and achieved a normal level of cognitive functioning. The definition of OO that Fein and colleagues suggest [3] foresees the absence of the typical symptoms of autism (deficits in the social, communicative, and affective spheres and restricted and repetitive behaviours and interests) and the presence of an average IQ, although other vulnerabilities may still be present, for example, in executive functioning or in anxious or depressive symptoms.

Over the years, there has been much debate over the role of adequate linguistic and intellectual functioning in autism. For some authors, the IQ does not represent a predictor of possible diagnostic changes from the autism classification, as reported in a systematic review [5], in which the findings showed that in only 4 out of 11 randomized studies analyzed the initial IQ assessed was associated with OO.

Kelley et al. [6] studied children between 5 and 9 years of age with a prior diagnosis of autism who were no longer showing significant symptoms and compared them to a group of typically developing children matched by age, sex and productive vocabulary. The results showed that the children in the two groups achieved the same level of language skills, but the children with a prior diagnosis of autism continued to present difficulties in the semantic and pragmatic aspects of language (theory of mind, construction of narratives, etc.).

In a recent study of highly functioning adults with autism, Otsuka et al. [7] investigated the combination of verbal and emotional competences as a predictor of good social and adaptive coping skills and found that emotional competence represents an important “mediator” of the relationship between verbal skills and positive outcomes.

Therefore, from a clinical perspective, it would be interesting to acquire more knowledge not only on cognitive factors as predictors of positive outcomes but also on the emotional and relational aspects of children at the time of the first assessment. In this way, the research results could increasingly support the planning of targeted and personalized treatment and identify the therapeutic approaches that could be most useful and effective for children with specific characteristics at the time of treatment beginning.

A growing number of therapeutic approaches exists for the management of autism, in particular to lessen the impact of symptoms on children’s functioning [8, 9]. For example, Early intensive behavioral interventions for young children with ASD (e.g. Lovaas approach, Early Start Denver Model; the TEACHH, the Applied Behaviour Analysis) currently represent the most well-known and studied methods for ASD management [8, 10]. There is also another group of research on naturalistic and psycho-developmental approaches, such as *DIR-Floortime* model (Developmental, Individual-Difference, Relationship-based model [11];), or *DERBBI* model (Developmental, Emotional Regulation and Body-Based Intervention [12];); these models presuppose that the communicative, cognitive, emotional and social skills are acquired through meaningful relationships and often through body-mediated interactions.

Finally there is an increasing number of studies have begun to examine the beneficial effects of the inclusion of animals in both recreational and therapeutic interventions [Animal Assisted Interventions, which demonstrate the usefulness of these therapies included in multidisciplinary treatments [13, 14]. However, despite the large number of therapeutic approaches, at present neither proven therapies nor preventive measures exist for the universal treatment of autism.

In this study we retrospectively investigated the predictive value of emotional-relational factors (emotional contagion and understanding of the intentions of others) in children with a diagnosis of autism who showed OO after at least 2 years of therapy, thus by achieving the lack of impairments in social and communication skills and in repetitive behaviours.

The purpose of the study is to verify the presence of emotional-relational factors that predict OO, including children’s level of functional or symbolic play since this aspect is related to executive functioning [15] and constitutes a learning factor associated with greater cognitive and communicative development [16, 17].

## Method

### Participants

The study included 40 children who had received an initial diagnosis (T0) at 35.3 months ±10.4 months (range: 21–66 months; median: 34 months). At the time of diagnosis, all children showed severe impairment of verbal language compared to chronological age.

At T0, 62.5% of the children were older than 30 months and fell into the ASD classification. A total of 37.5% were younger than 30 months and fell into the ASD Risk classification (*N* = 1, Mild Risk; *N* = 4, Medium Risk; *N* = 10, High Risk) as identified by the ADOS-2 and the DSM-5 (APA, 2013).

The children were divided into two groups: the ASD-ASD group included children (*N* = 25) with a confirmed diagnosis of ASD 2 years after the first assessment, and the ASD-OO comparison group included children (*N* = 15) who no longer met the criteria for an autism classification after 2 years (Table 1).

**Table 1:** Description of the sample at T0 (*N* = 40)

	ASD-OO group (***N*** = 15)	ASD-ASD group (***N*** = 25)	***P***
Age, in years, mean (SD)	2.9 (1.0)	3.0 (0.8)	.69
Sex, (% male)	12 out of 15 (80%)	18 su 25 (72%)	.57
ADOS-2, classification at T0			
Autism Spectrum, N (%)	8 out of 15 (53.3%)	17 su 25 (68%)	.20
Mild Risk, N (%)	1 out of 15 (6.7%)	0	
Middle Risk N (%)	3 out of 15 (20%)	1 su 25 (4%)	
Severe Risk, N (%)	3 out of 15 (20%)	7 su 25 (28%)	
Siblings, N (%)	10 out of 15 (66.6%)	20 su 25 (80%)	.34
Siblings with neurodevelopmental disorders, N (%)	3 out of 10 (30%)	7 su 20 (35%)	.78

In the ASD-ASD group, 68% of children (*N* = 17) received the first diagnosis at an age of over 30 months and 32% (*N* = 8) at an age of less than 30 months.

In the ASD-OO group, 53.3% of children (*N* = 8) received the first diagnosis at an age of over 30 months and 46.7% (*N* = 7) at an age of less than 30 months.

The percentage of males did not differ between the two groups (ASD = 82% vs. OO = 72%) (chi square = 0.32; *p* = .57). The distribution of the sample is in line with the most recent estimates, which indicate a prevalence of males to females in the ratio 4:1 [18, 19].

Most of the children (37 of 40) came from Italian families of average sociocultural background, and 25% of the sample were only children (*N* = 10). With respect to the presence of siblings with neurodevelopmental disorders, there were no differences between children in the ASD-OO group (30%) and the ASD-ASD group (35%), and the presence of symptomatic siblings appeared equally distributed.

### Procedure

Participants were recruited from the Institute of Ortofonologia. In this study, we enrolled 40 children admitted between September 2015 and March 2016 for an autism spectrum disorder clinical suspicious. The clinical and psychodiagnostic assessment was conducted by a team of qualified clinicians (with at least 5 years of experience in the field of autism) consisting of psychologists/psychotherapists, neurologists, paediatricians, child neuropsychiatrists, and rehabilitation therapists. The diagnosis of autism was based on the DSM-5 criteria [20]. In addition to clinical observations, the children were administered the Autism Diagnostic Observation Schedule (ADOS-2) [21] and were interviewed with their parents through questionnaires and rating scales (all assessments were video-recorded).

Once the diagnosis was confirmed, the children were included in the same model of autism therapy by the Institute Ortofonologia. The experts who conducted the assessments of the children and administered the ADOS-2 were different than those administered the child’s therapy.

Children with the following characteristics were not included in the research: (a) neurological disorders or focal neurologic signs; (b) severe sensory deficit (blindness and deafness); (c) history of severe birth injuries such as asphyxia, head trauma or epilepsy; and (d) other ASD pathogenic causes identified through high-resolution karyotype examination, DNA analysis for Fragile X, or positive screening tests for inborn errors of metabolism.

All children in the study group at T0 (before starting therapy) and at T1 (after 2 years of therapy) were administered the ADOS-2 for the evaluation of autistic symptoms and symbolic play, the TCE for measuring empathy and emotional contagion, the Leiter-R or WPPSI-III for assessing cognitive strategies, and the UOI procedure for measuring the understanding of others’ intentions.

### Instruments

#### ADOS-2 - autism diagnostic observation schedule - second edition

The ADOS-2 allows a standardized and semi-structured evaluation of the aspects of communication and social interaction (SA), restricted and repetitive behaviours (RRB) and playful/imaginative use of the material, involving a series of activities that directly elicit behaviours linked to the diagnosis of autism spectrum disorder [21].

It consists of several modules. Those used in this research were as follows.

- The Toddler Module is used for children between 12 and 30 months of age who do not consistently use phrase speech. This module provides scores that describe different clinical risk ranges for autism (none or low risk: scores from 0 to 9; moderate risk: from 10 to 13; high risk: greater than 13) to allow the clinician to quantify and formalize a clinical impression to avoid a formal classification that may not be appropriate in this age group.

- Module 1 is administered to children aged 31 months and over who use little or no phrase speech. It consists of a series of structured activities aimed at investigating aspects related to the area of social affect and restricted and repetitive behaviours. Scores above 8 are indicative of autism spectrum disorder.

- Module 2 is administered to children under 30 months of age who use phrase speech but are not verbally fluent. It consists of activities of imaginative play and joint interaction and conversation. Scores above 7 are indicative of autism spectrum disorder.

#### Cognitive assessment

At T0, the Leiter International Performance Scale – Revised (Leiter – R) [22] was used to measure nonverbal IQ through nonverbal stimuli, which is useful in cases where subjects have verbal linguistic impairment. The IQ scores had a mean of 100 and a standard deviation of 15.

At T1, the WPPSI-III [23] was used for children who had matured to a sufficient language level. The WIPPSI-III is a multidimensional intellectual assessment that allows us to obtain a verbal, performance, and processing speed quotient as well as a total IQ. The IQ scores had a mean of 100 and a standard deviation of 15.

#### Play evaluation

Play skills were assessed through the activities contained in ADOS-2 [21]. The different play levels were coded with the attribution of scores (not included in the conversion algorithm for the ADOS-2 global score) according to the following levels of playful use of objects: symbolic play, which involves spontaneous, flexible and creative use of objects in representative mode (score 0); functional play, which involves the appropriate use of a variety of toys in a conventional way (score 1); and stereotypical play, which involves the stereotypical use of objects (score 2).

#### Emotional contagion test (TCE)

The TCE [24] enables the evaluation of emotional contagion from both a quantitative and a qualitative perspective (i.e., the presence or absence of affective attunement in the child) through the observation of the child’s emotional and behavioural response while facing a structured stimulus (video).

A response is considered absent if the child does not reproduce the motor pattern of the emotion and is evaluated with 0; a response is considered present if the child reproduces the motor pattern of the emotion and can be evaluated with 1, 2 or 3. A score of 1 indicates the principle of emotional contagion, with one emotional contagion response and 3/4 hints for the stimulus reproduction; a score of 2 represents emotional contagion with 2 to 4 responses of emotional contagion; and a score of 3 indicates empathy, when the child recognizes and is able to differentiate emotions.

The entire TCE evaluation procedure was video-recorded, and scores were assigned during the observation and then reconfirmed through video-recordings. The duration of administration and scoring was approximately 10 min.

The measures used to assess TCE were first administered by two experienced professionals who independently observed 20 autistic children. The interobserver reliability agreement was high (Cohen’s k = .90), indicating an excellent level of agreement.

#### Understanding of others’ intentions (UOI)

To evaluate the understanding of others’ intentions, a modified version of the intention condition of the Behavioural Enhancement Procedure was used [25]. The UOI procedure involves the use of 4 objects; for each object, the experimenter shows 3 failed attempts at target action. The children have not previously seen the objects used or the target action completed. They watch the experimenter attempt to perform the target action without success (for example, the experimenter has a wooden peg associated with a nylon noose that could be hung on the peg; he tries to perform the target action, but he fails). Then, the object is left on the table in front of the child, who is told, “Now it is your turn”. A score from 0 to 4 is assigned based on the number of tasks completed with reference to the 4 target actions. The number of target actions produced is coded as follows: 0 = absence of capacity (no task performed); 1 = low capacity (1 task performed); 2 = fair capacity (2 tasks performed); 3 = good capacity (3 tasks performed); 4 = excellent capacity (4 tasks performed).

The entire UOI evaluation procedure was video-recorded, and the coders assigned a score during the observation and then reconfirmed it through video-recordings. The duration of administration and scoring was approximately 5 min.

The measurements used to evaluate the UOI were administered by two experienced professionals (previously trained on 20 UOI evaluations). The interobserver reliability agreement was high (Cohen’s k = .92) and indicates an excellent level of agreement.

### Statistics

The measurements had a normal distribution, for which it was possible to use parametric statistics (ADOS scores: Asimm: .77; kurtosis: −.18. TCE: Asimm: −.38; kurtosis: −.79. UOI: Asimm: .66; kurtosis: − 1.00).

To evaluate the changes in the scores that the children obtained in the 2 years of therapy, a multivariate analysis of variance (MANOVA) was conducted for repeated measures. The effect size was calculated using the partial eta squared; thus, η^2^_p_ = 0.02 is considered a small effect, 0.13 a medium effect, and 0.23 a large effect [26, 27]. To analyse the changes over time of the measures based on the categorical variable of therapy, a chi-square analysis was conducted. Correlational analyses were conducted to evaluate the relationships between the scores obtained in the various measures. Binary logistic regression analysis was used to identify the predictive factors of OO and to calculate the odds ratio to estimate the probability that subjects with a greater number of positive indicators could achieve OO. The significance level was set at *p* < 0.05. All statistical analyses were performed using SPSS software version 19.0.

## Results

### Changes in autistic symptoms

At the time of initial diagnosis (T0), the ADOS-2 mean of infants < 30 months (Toddler Module) in the ASD-OO group was significantly lower (13.7 ± 4.4) than that of infants in the ASD-ASD group (20.0 ± 4.9) (*P* < .05; η^2^_p_ = .34). Similarly, the mean ADOS-2 score of infants > 30 months (Module 1) in the ASD-OO group was significantly lower (10.5 ± 2.9) than that of infants in the ASD-ASD group (20.1 ± 4.5) (*P* < .01; η^2^_p_ = .57).

After 2 years, at T1, it was found that the ADOS-2 scores of the Toddler Module significantly decreased in the ASD-OO group (*P* = 0.01; η^2^_p_ = .79) and remained unchanged in the ASD-ASD group (*P* = .28). Similarly, Module 1 ADOS-2 scores decreased significantly in the ASD-OO group (*P* = 0.05; η^2^_p_ = .17) and remained unchanged in the ASD-ASD group (*P* = .36).

The improvements that emerged in the ASD-OO group were not related to the age of the children (*P* = .22) or to the IQ score (*P* = .15).

In addition to the total ADOS-2 score, the scores of the Social Affect (SA) and Restricted and Repetitive Behaviours (RRB) subscales were analysed. Significant improvements emerged in the ASD-OO group but not in the ASD-ASD group (AS: time x group effect: *P* < .001; η^2^_p_ = .35; RRB: time x group effect: *P* < .01; η^2^_p_ =. 21).

### Changes in cognitive abilities

Regarding IQ, the initial mean of IQ scores was significantly higher in children in the ASD-OO group than in children in the ASD-ASD group (80.5 ± 20.6 vs. 64.3 ± 14.9) (*P* < .01; η^2^_p_ = .18). After 2 years, IQ scores significantly increased in both the ASD-OO group and the ASD-ASD group (0.0 = 0.01; η^2^_p_ = .42) (Table 2).

**Table 2:** Mean (SD) of variables measured in the OO (*N* = 15) and ASD (*N* = 25) groups, at intake (T0) and after 2 years (T1)

	ASD-OO group (***N*** = 15)		ASD-ASD group (***N*** = 25)	
	T0	T1	T0	T1
ADOS-2, score, mean (SD)				
Toddler Module (*N* = 15)	13.7 (4.4) ^*a, b*^	4.71 (1.06) ^*b*^	20.0 (4.9)	23.25 (.99)
Module 1 (*N* = 25)	10.5 (2.9) ^*a, b*^	5.62 (1.51) ^*b*^	20.1 (4.5)	18.06 (1.04)
Social Affect of ADOS-2, mean (SD)	9.9 (0.8) ^*a, b*^	4.0 (0.8) ^*b*^	16.0 (0.6)	15.2 (0.7)
Restricted repetitive behaviours of ADOS-2, mean (SD)	2.1 (0.4) ^*a, b*^	1.2 (0.3) ^*b*^	4.1 (0.3)	4.9 (0.3)
IQ, score, mean (SD)	80.5 (20.6) ^*a, b*^	99.3 (18.5) ^*b*^	64.3 (14.9) ^*a*^	83.0 (11.6)
UOI, score, mean (SD)	3.3 (1.1) ^*a, b*^	4.0 (0.2) ^*b*^	1.2 (1.4)	1.7 (1.4)
TCE, score, mean (SD)	1.9 (0.2) ^*a, b*^	2.9 (0.2) ^*b*^	1.0 (0.5)	1.3 (0.6)
Play, ADOS-2 score, mean (SD)	.5 (0.5) ^*a, b*^	.0 (0.0) ^*b*^	1.1 (0.6) ^*a*^	.9 (0.7)

*Legend*: a **=** significant difference between T0 and T1; b = significant difference between OO and ASD; *IQ* Intelligence Quotient, *UOI* Understanding of other’s intentions procedure, *TCE* Emotional Contagion Test

Again, the changes in the initial and final IQ scores were not related to the age of the children (*P* = .35) or to the initial ADOS-2 score (*P* = .15).

As shown in Table 2, after 2 years, the children in the ASD-OO group achieved scores that could be classified within the average range (99.3 ± 18.5), while those in the ASD-ASD group remained below the average (83.0 ± 11.6).

From a qualitative perspective, however, the wide variability of IQ scores in both groups should be emphasized. The high standard deviations suggest the presence of children who had severely deficient or normal scores at T1 in both the ASD-ASD group and the ASD-OO group.

### Changes in social and affective skills

Table 2 presents the mean of the scores on the UOI procedure for evaluating the capacity to understand others’ intentions. A score of 0 indicates a lack of capacity, 1 indicates low capacity, 2 indicates fair capacity, 3 indicates good capacity, and 4 indicates excellent capacity.

At T0, children in the ASD-OO group had significantly higher UOI scores than those in the ASD-ASD group (*P* < .01; η^2^_p_ = .36). After 2 years, improvements were only visible in ASD-OO children (*P* < .05; η^2^_p_ = .32).

As shown in Table 2, the children in the ASD-OO group started at T0 with mean scores indicative of a good capacity to understand others’ intentions (3.3 ± 1.1) and at T1 had scores classified as excellent capacity (4.0 ± 0.2); in contrast, the children in the ASD-ASD group remained stable and had absent or low capacity (T0: 1.2 ± 1.4 vs T1: 1.7 ± 1.4).

Similar results emerged in the scores of the TCE test, which assesses the presence of emotional contagion. A score of 0 indicates the absence of emotional contagion, 1 indicates a principle of emotional contagion, 2 indicates the presence of emotional contagion, and 3 indicates the presence of an empathic behaviour response.

At T0, children in the ASD-OO group had significantly higher TCE scores than ASD children (*P* < .01; η^2^_p_ = .21). Even after 2 years, only children in the ASD-OO group showed significant improvements (*P* < .001; η^2^_p_ = .53).

As shown in Table 2, the children in the ASD-OO group started at T0 with mean scores indicative of the presence of emotional contagion (1.9 ± 0.2), and at T1, scores that could be classified as empathic behaviour response (2.9 ± 0.2) were obtained. In contrast, the children in the ASD-ASD group remained stable with the principle of contagion (T0: 1.0 ± 0.5 vs. T1: 1.3 ± 0.6).

Finally, the level of play in children was assessed by observing and coding the playful use of objects and materials at the ADOS-2 assessment: stereotyped/cause-effect play has a score of 2, functional play has a score of 1, and symbolic play has a score of 0.

At T0, children in the ASD-OO group had significantly better play scores than those in the ASD-ASD group (*P* < .01; η^2^_p_ = .17).

After 2 years, significant improvements emerged in children in the ASD-OO group (*P* < .001; η^2^_p_ = .34) and in the ASD-ASD group (*P* < .05; η^2^_p_ = .09), although the low effect size (η^2^_p_) found in the ASD-ASD group defines this improvement as minimal. The chronological age of the child, entered as a covariate, was not significant (*P* = .08).

The children in the ASD-OO group started at T0 from mean scores indicative of functional and/or symbolic play (0.5 ± 0.5), and all reached T1 scores indicative of symbolic play ability (0.0 ± 0.0). In contrast, the children in the ASD-ASD group started at T0 from mean scores indicative of stereotyped and/or functional play (1.1 ± 0.6) and remained stable at the same level of play (0.9 ± 0.6).

### Combination of predictive indicators of optimal outcome

Considering the previous results, a combo variable was created within which the indicators of positive outcome were combined and categorized: presence/absence of normal IQ (> 85), presence/absence of emotional contagion or empathic behaviour, presence/absence of good or excellent capacity to understand intentions and presence/absence of complex functional and/or symbolic play.

Each of the 40 subjects of the sample was therefore attributed a value of the combo variable from 1 (presence of only one positive outcome indicator) to 4 (presence of all indicators).

The odds ratio (Exp-B) was calculated to estimate the probability that subjects with a greater number of positive indicators at T0 could achieve OO. As shown in Table 3, as the number of positive indicators increases, the possibility of an OO significantly increases. An odds ratio of 4.4 indicates a marked increase in the chances of re-entering the ASD-OO group.

**Table 3:** Binary logistic regression

	B	E.S.	Wald	df	Sig.	Exp(B)	95% CI for EXP(B)	
							Lower	Upper
N of positive indicators	1491	,455	10,738	1	,001	4,44	1821	10,836
*Constant*	*− 3934*	*1242*	*10,028*	*1*	*,002*	*,02*		

As shown in Table 4, a significantly different distribution of the number of positive predictors between the ASD-ASD and ASD-OO groups emerged from the chi-square analysis (chi-square = 22.14, *p* < .001).

**Table 4:** Contingency table and frequency distribution of positive indicators in the two groups ASD and OO

		ASD-ASD ***N*** = 25	ASD-OO ***N*** = 15	Total ***N*** = 40
N of positive indicators at T0 (IQ, TCE, UOI, PLAY)	No indicator	11	0	11
	1 indicator	4	1	5
	2 indicators	7	2	9
	3 indicators	3	4	7
	4 indicators	0	8	8

*Legend: IQ* Average Intelligence Quotient, *TCE* Presence of Emotional Contagion, *UOI* Presence of Understanding of Other’s Intentions; Play: presence of functional / Symbolic play

Eleven children who had no positive indicator at T0 remained in the ASD-ASD group; similarly, children (4 of 5) with 1 indicator and children (7 of 9) with 2 positive indicators remained in the ASD-ASD group. Of the 7 children who had 3 positive indicators at T0, 4 belonged to the ASD-OO group. The 8 children who had all 4 positive indicators all fell into the ASD-OO group.

The age of the children did not show correlations with the achievement of OO. Moreover, a qualitative analysis, as illustrated in Table 5, showed that symbolic play was the indicator that was consistently present among the children who achieved OO.

**Table 5:** Distribution of positive indicators in the two groups, ASD-ASD and ASD-OO

***N = 11; 0 Predictor***			***N = 5; One Predictor***			***N = 9; Two Predictors***		
**Group**	**Age**	**Predictor**	**Group**	**Age**	**Predictor**	**Group**	**Age**	**Predictors**
ASD-ASD	21 months	None	ASD-OO	21 months	PLAY	ASD-OO	32 months	PLAY, TCE
ASD-ASD	25 months	None	ASD-ASD	27 months	IQ	ASD-OO	32 months	PLAY, TCE
ASD-ASD	26 months	None	ASD-ASD	43 months	TCE	ASD-ASD	27 months	PLAY+ IQ
ASD-ASD	29 months	None	ASD-ASD	47 months	TCE	ASD-ASD	28 months	PLAY + TCE
ASD-ASD	31 months	None	ASD-ASD	50 months	TCE	ASD-ASD	30 months	TCE + UOI
ASD-ASD	32 months	None				ASD-ASD	35 months	PLAY + UOI
ASD-ASD	34 months	None				ASD-ASD	38 mesi	UOI + IQ
ASD-ASD	35 months	None				ASD-ASD	38 mesi	TCE + IQ
ASD-ASD	35 months	None				ASD-ASD	61 mesi	PLAY + IQ
ASD-ASD	38 months	None						
ASD-ASD	43 months	None						
***N = 7; Three Predictors***						***N = 8; Four Predictors***		
**Group**	**Age**	**Predictors**				**Group**	**Age**	**Predictors**
ASD-OO	27 months	PLAY+TCE + UOI				ASD-OO	25 months	ALL
ASD-OO	28 months	PLAY+UOI + QI				ASD-OO	26 months	ALL
ASD-OO	34 months	PLAY+TCE + UOI				ASD-OO	27 months	ALL
ASD-OO	66 months	PLAY+TCE + UOI				ASD-OO	27 months	ALL
ASD-ASD	34 months	PLAY+UOI + IQ				ASD-OO	33 months	ALL
ASD-ASD	38 months	PLAY+TCE + UOI				ASD-OO	36 months	ALL
ASD-ASD	52 months	PLAY +UOI + IQ				ASD-OO	50 months	ALL
						ASD-OO	52 months	ALL

*Legend*. *ASD* ASD-ASD group, *OO* ASD-OO group, *TCE* Presence of emotional contagion, *UOI* Presence of understanding of other’s intentions, *IQ* Intelligence quotient

## Discussion

Our data show that 15 out of 40 children, enrolled in this study and subjected to the same model of autism therapy at least for 2 years, no longer fell into the diagnostic ASD category based on the ADOS-2, DSM-5 and clinical criteria.

Children under 30 months of chronological age who passed the clinical cut-off for the ADOS-2 Toddler Module (children at risk for ASD) and children over 30 months who passed the ADOS-2 Module 1 clinical cut-off (children with ASD) were included in the study. Research over the past two decades has revealed that an ASD diagnosis can be considered very reliable at the age of 2 years [28–30], although many children do not receive a final diagnosis until they are older. This “latency to diagnosis” could pose a risk in that children may not receive timely help. In fact, the American Academy of Paediatrics [31] recommends that children undergo specific screening for ASD during regular medical visits at 18 and 24 months.

When studying samples under 30 months, the risk of misdiagnosis could increase; in the present research we tried to control this risk, including only children for an autism spectrum disorder clinical suspicious, from the local services; immediately afterwards, the children were assessed both directly and indirectly by a specialized team, as required by the DSM-5. Several studies have found that ASD can be reliably diagnosed in children under 3 years of ageby experienced, highly trained clinicians in specialty clinic and research settings [32, 33] and that the greatest accuracy in diagnosis of young children is achieved when using a standardized parent interview and a standardized observational measure in combination with clinical judgment [34].

In the present study, among the children under 30 months who fell into the ASD risk category at T0, after 2 years, 8 (out of 15) had a continuing diagnosis of ASD, while 7 achieved optimal outcomes (ASD-OO); thus, they lost the symptomatic characteristics (in terms of both ADOS-2 scores and clinical evaluation) that initially caused them to fall within a framework of high risk of ASD. This finding is consistent with the study of Helt et colleagues [35] who concluded that between 3 and 25% of individuals with ASD eventually lost their diagnosis. From a therapeutic perspective, all the children continued the treatment and monitoring path in the following years, as suggested by the main paediatric associations [31].

At T0, the children in the ASD-ASD and ASD-OO groups were homogeneous for chronological age, sex, and family features and were all characterized by a poor linguistic vocabulary. For this reason, language skills were not included among the predictive variables investigated in this research.

The most relevant result that emerged from the research is that the two groups instead differed from the intake (T0), in all socio-cognitive variables measured; indeed the children in the ASD-OO group initially had a higher IQ than those in the ASD-ASD group, lower severity of autistic symptoms, greater understanding of intentions, more emotional contagion, and better quality of play. Therefore, these factors have been investigated as probable predictors of optimal outcome.

Regarding the IQ, although the ASD-OO children had higher initial scores than the ASD-ASD group, the children in both groups showed improvements over time in cognitive functions (IQ) regardless of the chronological age factor and the initial autism severity score. This increase can be interpreted as the emergence of previously unexpressed potential [36]; meaning that, when symptomatology decreases, planning and organization skills can emerge more easily [25]. It should also be noted that the children included in this study followed a rehabilitation path based on a developmental approach [12] that focuses its intervention on mainly psycho-physical rather than cognitive activities, so the improvement in cognitive functioning is not a consequence of specific enhancements.

These data seem to be in line with the results of Sutera and colleagues [37], who studied children who no longer had a diagnosis of autism after 2 years of treatment (the authors do not specify which treatment) and found adequate profiles in adaptive behaviour, visual reception, motor skills, language and IQ.

Regarding the socio-affective variables, and in particular to emotional contagion, the results of this research suggest that from the moment of the first diagnosis, children who undergo a positive symptomatic evolution (ASD-OO) have a better capacity for emotional contagion, a skill that improves over time until they reach normal levels.

Additionally, a recent study [38] highlighted the presence of emotional contagion in children with autism, especially in conditions of familiarity with the interlocutor. The authors note that while children with typical development can show emotional contagion in all conditions, children with ASD are strongly influenced by the familiarity of the stimulus. If they are exposed to familiar figures (for example, parents or teachers), they are able to show a quality of emotional contagion similar to that of their peers with typical development.

In the literature, however, there are still few studies on social and affective factors as predictors of OO. For this reason, one of the variables that we considered was the presence of emotional contagion; perceiving the emotions of others generally leads to an empathic concern based on both emotional and cognitive processes. The precursor of empathy is emotional contagion, the phenomenon whereby a person’s emotions and related behaviours directly trigger similar emotions and behaviours in other people. Emotional contagion is important for personal relationships because it fosters emotional synchrony between individuals [39]. Reduced empathy has long been regarded as a contributing factor to social difficulties in individuals with ASD. In contrast to these definitions, a recent study [40] conducted with high-functioning adolescents with ASD who were given a functional MRI while watching short video clips of people suffering pain argued that there were no significant differences in brain activation between individuals with ASD and typically developing individuals. The authors therefore suggested that the mechanisms involved in emotional empathy may be preserved in high-functioning ASD individuals.

As for the ability to understand the intentions of others, which is usually present in typically developing children from 18 months of age [41, 42], it was considered as another precursor of cognitive empathy. The data of the present research describe the presence, from the first diagnosis, of these abilities in children in the ASD-OO group but not in children in the ASD-ASD group.

Understanding intentions involves recognizing that physical actions depend on the goals and intentions of the actor. Studies that have investigated this component in ASD children often have not found impairments in the ability to understand intentions in children between the ages of 2 and 5. Despite this, children’s performance was lower when the understanding of intentions also involved social sharing, as in the case of shared attention [43, 44].

These findings offer a new perspective on whether emotional contagion, the ability to understand the intentions of others and the prerequisite of empathy in general, is neither deficient nor intact in children with ASD but whether they can demonstrate it depending on the contexts and relationships in which they are involved [38].

With respect to the quality of the play, and in particular to the presence of symbolic play, in which the child was required to treat an object or a situation as if it was something else (eg, using a banana as a telephone) the results of our study suggest that at T0, the children of the ASD-OO group had a qualitatively better play (mostly functional or symbolic) than children of the ASD-ASD group (mostly stereotyped or cause-and-effect). Two years later, significant improvements emerged in the children of both groups, regardless of chronological age. In literature, symbolic play has been described as an autism-specific deficit as children with other developmental disorders tend to show comparable amount of play with typically developing children [45], but some authors have found that symbolic play was not significantly associated with verbal or non verbal ability and weakly correlated with executive functions measures, while it was associated with theory of mind measures [46]. They argued that the observed deficit in symbolic play is due to deficit in cognitive mechanism rather than the inability to understand verbal instructions and produce verbal responses in the play trails. A better understanding of the underpinning impairments associated with symbolic play thus contributes to the development of effective intervention as well as play development in children with typical development.

Finally, regarding the main objective of our study, namely, to identify a combination of predictive indicators of positive outcome, these indicators were IQ, emotional contagion, understanding of others’ intentions and level of play achieved. The results suggest that, taken individually, none of the factors considered are predictive of OO. The mere presence of one or two predictive indicators at the time of diagnosis is related to a rare possibility that the child may no longer show autistic symptoms after at least 2 years of therapy. To have a good chance (57%) of reaching an OO, at least 3 predictive indicators must be present. Only children in whom the coexistence of all 4 predictive factors was identified at the time of diagnosis showed OO in 100% of cases. If we consider that, with the exception of IQ, all the indicators have a social-affective nature, this seems to be in line with research in the neuroscientific, psychodynamic and cognitive fields [47–51], which argues that at preschool age, a therapeutic path for autism spectrum disorders must be considered fundamental for intervening in the affective-relational components that represent the foundation for the expression of cognitive and, ultimately, communicative potentialities. The usefulness of early intervention has its roots in the concept of neuroplasticity, that is, the biological ability of the central nervous system to undergo maturation, change structurally and functionally in response to experience and adapt following an injury.

In the healthy developing brain, neuroplasticity exhibits a heterochronous cortex-specific developmental profile and is heightened during “critical and sensitive periods” of pre- and postnatal brain development that enable the construction and consolidation of experience-dependent structural and functional neural connections.

## Conclusions

Professionals who deal with autism in both the diagnostic and intervention phases have long questioned the different developmental trajectories over time of children with ASD and ways of individualizing treatments and therapeutic projects. As our data suggest, it is important to pay attention in both in the diagnostic and prognostic phases to emotional-relational, communicative, and social factors, which could support clinicians in defining a more complete diagnostic framework and in planning a more personalized therapeutic path. Through a retrospective analysis, we found that during the first assessment, the coexistence of emotional contagion, understanding of intentions and functional and/or symbolic play can represent prognostically positive factors for OO. The instruments used in this study can be rapidly administered since they require 5 min (UOI and Play) to 10 min (TCE) and are therefore suitable for short observation sessions in paediatric patients. During the check-up that clinicians (paediatricians, neuropsychiatrists and psychologists) usually perform with infants and children during the first year of life, it is now common practice to pay attention to the neurobehavioural profile, such as sucking/swallowing reflexes, the rhythms of sleep, body postures and gaze patterns. Approximately 18 months later, further observations are added, such as the presence of gaze fixity, repetitiveness or dispersiveness during free activities and the absence of deictic gestures or joint attention. Therefore, relatively simple and increasingly refined tools that add information to the clinician’s observational picture represent an important opportunity for prognostic purposes. Currently, among the indirect assessment tools, for example, paediatricians have a parent-report screening tool such as the Modified Checklist for Autism in Toddlers, Revised (M-CHAT-R [52];), a useful for children aged 16 to 30 months of age.

Without prejudice to the mandatory nature of specific assessments for diagnostic purposes (for example, through the ADOS-2, the ADI and intellectual assessment scales), for which a second-level assessment is usually referred to the multidisciplinary team, we believe that it may be useful to integrate activities and proposals that highlight the presence of social-relational skills, such as the level of play achieved, the child’s ability to understand what others are about to do and sensitivity to the emotions manifested by others. A properly trained paediatric specialist can acquire the skills to conduct an important and qualified screening in a short time. Early diagnosis and therapeutic intervention, as demonstrated in this study, are essential for the sustainability of the system and for the optimization of treatment.

The sharing of the diagnostic path between specialists supports comparison not only of “concerns” with respect to the child’s development but also of the resources available to the child and alternatives to be offered to parents, especially during the first years of the child’s life, which represent a unique “window of opportunity” for the child’s “plastic” development. The possibility of early identification of indicators capable of guiding therapeutic paths and various prognostic opportunities represents a significant enhancement of the role of the paediatrician in providing appropriate diagnoses, the correct use of specialist services, and the ability to promptly modify potentially worsening evolutions and thus to ensure the sustainability of the health system.

A limitation of this research concerns the short period (2 years after the first diagnosis) of functional re-evaluation. Since all the children who participated are included in a four-year treatment plan, it will be interesting to monitor any changes after another 2 years.

In addition, it would be useful to increase the number of subjects of different age groups, in order to study with more accuracy the effect of early diagnosis (< 30 months) compared to a later diagnosis.

## Data Availability

The data that support the findings of this study are available from Institute of Ortofonologia, but restrictions apply to the availability of these data, which were used under license for the current study, and so are not publicly available. Data are however available from the authors upon reasonable request and with permission of Institute of Ortofonologia.
